# A Human Anti-Toll Like Receptor 4 Fab Fragment Inhibits Lipopolysaccharide-Induced Pro-Inflammatory Cytokines Production in Macrophages

**DOI:** 10.1371/journal.pone.0146856

**Published:** 2016-01-19

**Authors:** Maorong Wang, Wenkai Zheng, Xuhui Zhu, Jing Xu, Binggang Cai, Yiqing Zhang, Feng Zheng, Linfu Zhou, Zhiguo Yang, Xin Zhang, Changjun Wang, Shinan Nie, Jin Zhu

**Affiliations:** 1 Institute of Liver Disease, Nanjing Jingdu Hospital, Nanjing 210002, China; 2 Huadong Medical Institute of Biotechniques, Nanjing 210002, China; 3 Department of Pathology, Key Laboratory of Antibody Technique of the Ministry of Health, NJMU, Nanjing 210029, China; 4 Anhui Medical University Affiliated with Bayi Clinical College, Nanjing 210002, China; 5 Department of Emergency Medicine, Jinling Hospital, Medical School of Nanjing University, Nanjing 210002, China; 6 Department of Respiratory Medicine, The First Affiliated Hospital of Nanjing Medical University, Nanjing, China; SRI International, UNITED STATES

## Abstract

The results of clinical and experimental studies suggest that endotoxin/toll-like receptor 4 (TLR4)-mediated proinflammatory and profibrotic signaling activation is critical in the development of hepatic fibrosis. However, studies examining the role of specific TLR4 inhibitor are still lacking. The present study was aimed to prepare a human anti-TLR4 Fab fragment, named hTLR4-Fab01, and to explore its immune activity. We screened the positive clone of anti-human TLR4 phagemid from a human phage-display antibody library using recombinant TLR4 protein, which was used as template cDNA for the amplification of variable regions of the heavy (V_H_) chain and light chain (V_L_), then coupled with highly conserved regions of the heavy chain domain 1 (C_H_1) and the light chain (C_L_), respectively. Thus, the prokaryotic expression vector pETDuet-1 of hTLR4-Fab01 was constructed and transformed into *Escherichia coli* (*E*. *coli*) BL21. The characteristic of hTLR4-Fab01 was examined by SDS-PAGE, Western blotting, ELISA, affinity and kinetics assay. Further, our data demonstrate that hTLR4-Fab01 could specifically bind to TLR4, and its treatment obviously attenuated the proinflammatory effect, characterized by less LPS-induced TNF-α, IL-1, IL-6 and IL-8 production in human macrophages. In conclusion, we have successfully prepared the hTLR4-Fab01 with efficient activity for blocking LPS-induced proinflammatory cytokines production, suggesting that the hTLR4-Fab01 may be a potential candidate for the treatment of hepatic fibrosis.

## Introduction

Hepatic fibrosis is characterized by wound-healing stimulation and inflammatory response to chronic liver injury. Chronic inflammatory would promote scar tissue to form, known as fibrosis, which results from persistent chronic liver injuries and inflammation, such as viral hepatitis, chronic alcohol consumption, autoimmune disease and metabolic disorder [1]. Prolonged liver injury results in hepatocyte damage, which triggers activation of Kupffer cells (KCs) and hepatic stellate cells (HSCs) [2]. Following bacterial or viral invasion, activated KCs produce a variety of proinflammatory cytokines, such as tumor necrosis factor (TNF)-α, interluekin-1β (IL-1β), interluekin-6 (IL-6) and interluekin-8 (IL-8) that promote the activation of HSCs [3]. During activation, HSCs proliferate and express excessive α-smooth muscle actin (α-SMA), and produce large amounts of extracellular matrix components (ECM), including type I collagen and fibronectin [4]. Because KCs are recognized as the primary cellular source of inflammatory cytokines in chronic liver diseases, and play a critical role in the development and maintenance of liver fibrosis, targeting their activation has become a key point in treating liver fibrosis [5].

Toll-like receptors (TLRs) are one of the most important pattern recognition receptors that can detect invading pathogens through recognition of pathogen-associated molecular patterns (PAMPs) such as LPS and initiate protective innate immune responses [6]. Upon recognition of LPS, TLR4 activates a variety of proximal signaling pathways, such as the MyD88 and/or TRIF-dependent signaling cascades and leads to production of proinflammatory cytokines and type I interferon by activating MAPKs, NF-κB, and IRF-3 pathways [7]. In the liver, TLRs are expressed in many different cell types including KCs, hepatocytes and HSCs [8]. Recent data suggests that Serum LPS levels are obviously elevated in patients with chronic hepatitis and cirrhosis [9–10] Specifically, TLR4 is predicated to be involved in pathogenesis of chronic hepatic inflammation, injury and fibrosis [11]. The fundamental role of TLR4 activation in the development of hepatic fibrosis has been shown by using TLR4 mutant mice as well as antibiotic treatment. Additionally, it has been demonstrated that TLR4-mutant mice critically attenuated fibrosis in the bile duct ligation (BDL) model and antibiotic treatment suppresses liver fibrosis [12]. These facts suggest a strong contribution of LPS-TLR4 interaction in the development of liver fibrosis. As mentioned above, KCs are direct targets of LPS in vitro and in vivo. Therefore, the interruption of TLR4 signaling could be as a target to suppress the activation of KCs and disrupt the progression of liver fibrosis.

Although full activation of TLR4 pathway is essential for initiating the innate immune response and enhancing adaptive immunity to eliminate invading microbial pathogen, excessive activation of TLR4 signaling may cause immune disorders such as inflammatory disease and sepsis shock. Thus, TLR4 signaling is strictly regulated to maintain the immunological balance. Recently, some negative regulators of TLR4-triggered inflammatory innate response have been identified, which may contribute to fine-tune LPS induced immune reaction [13–15]. However, new negative regulators (even blocking agents) of the TLR4-triggered inflammatory response should be further found in the immune cells, especially for treatment of inflammatory disease and hepatic fibrosis.

In the present study, we prepare a human anti-TLR4 Fab fragment (hTLR4-Fab01), and to examine its immune activity in human macrophages, which are differentiated from THP-1 by stimulation with phorbol 12-myristate 13-acetate (PMA) [16]. Differentiated THP-1 macrophages have been well established as an in vitro model of human macrophages in studies of macrophages involvement in inflammatory disease [17]. Our study is intrigued to evaluate the role of hTLR4-Fab01 in human macrophages, so as to provide a meaningful target for preventing hepatic fibrosis in clinics.

## Materials and Methods

### Cell Culture and Differentiation

THP-1 cell line was obtained from the cell bank of Shanghai Institute of Biochemistry and Cell Biology (the Chinese Academy of Science, Shanghai, China). The cells were maintained as describe previously [18]. Briefly, THP-1 cells were cultured in RPM 1640 medium (Lonza, MD, USA) containing 10% fetal bovine serum (FBS) (Gibco, NY, USA) and 2 mmol/L L-glutamine, maintained at 37°C in a humidified 5% CO2 incubator. THP-1 cells (2×10^5^ cells/ml) were differentiated using 10nM phorbol 12-myristate 13-acetate (PMA) (Sigma-Aldrich, MO, USA) for 48–72 hours.

### Phage Library and Helper Phage

A human naive Fab phage library for TLR4 was generated as described previously [19]. Before the first-round panning, the phage library was titrated and 1×10^13^ phage clones were collected for panning.

### Phage ELISA

Phage ELISA was performed as described previously with modification [20]. Briefly, phage clones from the *E*. *coli* XL1-Blue infected by the seventh round of eluted phage were randomly picked up and grown in 1mL super broth (SB) medium containing 100 μg/ml of ampicillin and 1% glucose. VCSM13 helper phage (1×10^9^) was then added to each vial. Fifty microliters of supernatant from each vial was added to each well of 96-well plates coated with 100 ng extracellular domain of TLR4 that had been preblocked with 5% milk blocking buffer. After incubation and washing, 50 μL of horseradish—peroxidase (HRP)-conjugated anti-M13 antibody (Amersham Pharmacia Biosciences, NJ, USA; 1: 5,000 diluted in blocking buffer) was added to each well, followed by incubation with 50 μL of HRP substrate solution (Pierce, IL, USA). The absorbance value at 450 nm was read by Multiskan Spectrum Microplate (Thermo Electron Corporation, MA, USA). The phage ELISA assays were repeated for three times. One of the triple positive clones with the highest absorbance was chosen for further evaluation.

### Construction of the Vector for the Expression of hTLR4-Fab01

The total RNA was extracted from positive clones by the TRIzol Reagent (Invitrogen, CA, USA), and cDNA was synthesized using PrimeScript RT reagent (Takara Company, Dalian, China) according to the manufacturer’s protocols. The variable regions of the heavy (V_H_) and light chains (V_L_) were amplified by PCR with degenerate primers. The conserved regions of the heavy chain domain 1 (C_H_1) and the light chain (C_L_) were amplified from pcomb3XTT, which was kindly provided by the Barbas laboratory (Scripps Research Institute, USA). PCR products of V_H_ and V_L_ were purified and then clone into pETDuet-1 at *Nde* I/*Xho* I and *Nco* I/*Hind* respectively. The heavy chain Fd and light chains L were amplified from V_H_ combined with C_H_1 and V_L_ combined with C_L_ using a forward primer L1 or F1 in combination with a reverse primer L4 or F4) respectively. The primers were described in Table 1. The PCR products of Fd and L were cloned into pETDuet-1 at *Nde* I/*Xho* I and *Nco* I/*Hind* III respectively. The recombinant vectors pETDuet-1/hTLR4-Fab01 were sequenced and further analyzed using the VBASE2 database (http://www.vbase2.org/).

**Table 1:** Primers used for the construction of the hTLR4-Fab01 gene.

Primer name	DNA sequence
Heavy chain variable region forward primer	
HuIgVH5'-A	GGGAATTCATGGACTGGACCTGGAGGRTCYTCTKC
HuIgVH5′-B	GGGAATTCATGGAGYTTGGGCTGASCTGGSTTTYT
HuIgVH5′-C	GGGAATTCATGRAMMWACTKTGKWSCWYSCTYCTG
Heavy chain variable region reverse primer	
HuIgMVH3′-1	CCCAAGCTTAGACGAGGGGGAAAAGGGTT
Light chain variable region primer	
HuIgλVL5′-A	GGGAATTCATGRCCTGSWCYCCTCTCYTYCTSWYC
HuIgλVL3′-1	CCCAAGCTTGAAGCTCCTCAGAGGAGGG
Heavy chain variable region primer	
F1	CATATGCAGGTGCAGCTGGTGCAGTCT
F3	TGGGCCCTTGGTGGAGGCTGAGGAGACGGTGACCAGGG
Light chain variable region primer	
L1	CCATGGAGCTCGTGGTGACGCAGCCG
L3	CAGCCTTGGGCTGACCTAGGACGGTCAGCCTGG
Constant region CH primer	
F2	CCCTGGTCACCGTCTCCTCAGCCTCCACCAAGGGCCCA
F4	CTCGAGTTAAGAAGCGTAGTCCGGAACGTC
Constant region CL primer	
L2	ACCAGGCTGACCGTCCTAGGTCAGCCCAAGGCTG
L4	AAGCTTTTATGAACATTCTGTAGGGGCCACT
Heavy chain Fd primer	
F1	CATATGCAGGTGCAGCTGGTGCAGTCT
F4	CTCGAGTTAAGAAGCGTAGTCCGGAACGTC
Light chain primer	
L1	CCATGGAGCTCGTGGTGACGCAGCCG
L4	AGCTTTTATGAACATTCTGTAGGGGCCACT

### Expression and Purification of hTLR4-Fab01

A single clone was reinoculated in LB medium containing 100 mg/ml of ampicillin, induced by 1 mmol/L isopropyl β-D-thiogalactopyranoside (IPTG) at 37°C and harvested 24 hours later. Both bacteria lysate and sonicated supernatant were detected by SDS-PAGE with Coomassie blue staining. The soluble hTLR4-Fab01 was purified from the periplasm by immobilized metal affinity chromatography (IMAC) using His-trap Lambda Fab Select column (GE healthcare, Madison, WI, USA) according to the manufacturer’s instructions. The purity of the hTLR4-Fab01 was analyzed by SDS-PAGE (12%) or native-page (Bio-Rad, CA, USA) with Coomassie Blue staining.

The endotoxin concentration during the Fab preparation was examined with ToxinSensor^™^ Chromogenic LAL Endotoxin Assay Kit (Genscript, Nanjing, China). The hTLR4-Fab01 solution was purified with ToxinEraser^™^ endotoxin removal resin (Genscript, Nanjing, China) The final endotoxin level of Fab solution was decreased to less than 0.1 EU/ml.

### Western Blot

The expression of hTLR4-Fab01 in *E*. *coli* were performed by Western blot as described previously [21]. Typically, bacteria lysate was prepared supplemented with a proteinase inhibitor cocktail (Roche, IN, USA). Protein concentration was examined using a bicinchoninic acid (BCA) Protein Assay kit according to the manufacturer’s instruction (Pierce, IL, USA). The protein from whole-cell lysate were separated by 10% SDS-PAGE and transferred to Nitrocellulose membrane (Bio-Rad, CA, USA). To determine the antigenicity of the purified Fab fragment, the membrane was incubated with HRP-conjugated goat anti-human Fab specific antibody (Santa Cruz Biotechnology, CA, USA) for 1 h at room temperature. The bands were visualized using DAB Chromogenic Reagent (Boster company, Wuhan, China) according to the manufacturer’s instructions.

### Affinity Determination of the hTLR4-Fab01

The affinity of hTLR4-Fab01 was determined by non-competitive ELISA [22]. Briefly, 96 wells plate was coated at 4°C with recombinant human TLR4 (R&D Systems, MN, USA) at 10 μg/ml overnight. the plate was blocked with 5% BSA, then serial concentrations of the hTLR4-Fab01 were added (3 replicated wells for each concentration) as the primary antibody. HRP-conjugated anti-human Fab specific antibody was used as the secondary antibody. Commercial anti-TLR4 antibodies (Abcam, MA, USA) were used as positive controls. The absorbance at 450 nm was detected and plotted as a histogram with Excel (Microsoft, WA, USA).

### SPR analysis of hTLR4-Fab01

Analyses were performed with the Biacore X100 Plus Package evaluation software, version 1.1 [23]. Briefly, basing on the isoelectric point and in accordance with the protocol for Biacore X100 Surface Plasmon Resonance (SPR) system (GE, Sweden) optimization of coupling conditions, sodium acetate was chosen as the coupling dilute buffer. After diluting the sample with the buffer solution to 30 μg/ml, it was coupled to a CM5 chip. The coupling level was preset at 1500 RU. The sample was treated with a running buffer containing different concentrations of hTLR4-Fab01. The injection time was set to 180 s, the dissociation time was set to 15 min, and 50 mM Gly-HCl (pH = 1.7) was used as the regeneration buffer. All experiments were performed in triplicate.

### Flow Cytometry

Specific binding of the hTLR4-Fab01 to TLR4 was determined by FACS analysis. Briefly, THP-1 transformed macrophages were fixed using BD Cytofix/Cytoperm buffer (BD Biosciences, CA, USA) for 10 min, blockaded with 1% FBS for 1 h, and then incubated for 1 hour with hTLR4-Fab01. Cells were washed with PBS and analyzed using an LSR II flow cytometer (BD Biosciences, CA, USA). Proper isotype controls were included [24].

### Cytokines Detection

TNF-α, IL-1β, IL-6, and IL-8 in the supernatants were measured with ELISA kits (R&D Systems, MN, USA) according to the manufacturer’s instructions. Briefly, THP-1 transformed macrophages were treated with serum-free medium containing different concentration of hTLR4-Fab01 and incubated for 2 h. Anthrax chimeric Fab antibody was used as negative control [25]. Then, cells were cultured in complete medium and stimulated with 100 ng/ml LPS for 8 h. The supernatant was harvested and examined.

### Statistical analysis

The statistical significance of comparisons between two groups was determined with Student’s *t* test. Mean ± SD was calculated for each group. A value of P <0.05 was considered to indicate statistical significance.

## Results

### Screening of strains positive for hTLR4-Fab01 and construction of the expression vector

**Fig 1:**
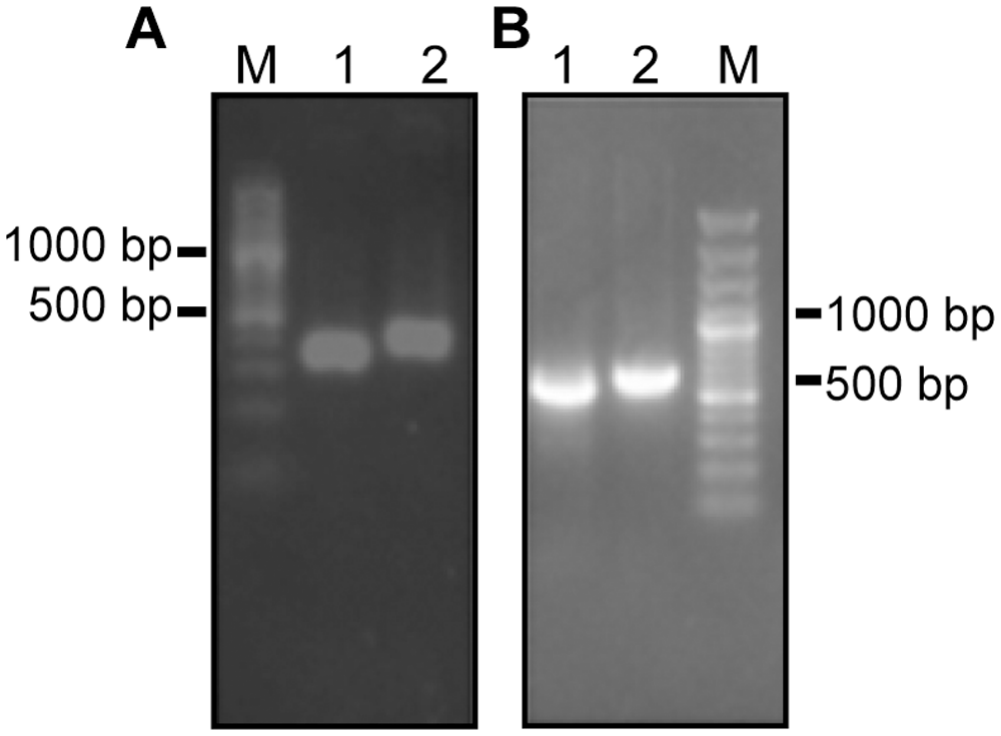
Screening of strains positive for hTLR4-Fab01. (A) PCR products of positive phages. Lane 1, V_L_; lane 2, V_H_; lane M, DNA marker. (B) The heavy chain Fd and light chain L were spliced. Lane 1, L chain; lane 2, Fd chain; lane M, DNA marker.

With five rounds of affinity panning, 80 clones were selected and identified using phage ELISA. When the Positive/Negative was greater than 4, phages were deemed to be positive. Eleven positive phage clones were obtained, amplified, and confirmed by sequencing. All positive phage clones possessed the same F_V_ sequence by aligning. After analyzing with the VBASE2 database, the V_L_ of F_V_ was the lambda chain. As expected, the length of V_L_ and C_H_1 products were about 350 bp, while V_H_ and C_L_ were about 400 bp (Fig 1A). As shown in Fig 1B, heavy chain Fd (≈800 bp), light chain L (≈750 bp) were amplified by RT-PCR. Sequence analysis illustrated that the DNA sequence of the Fd and L fragments have been successfully inserted into the pETDuet-1 prokaryotic expression plasmid, which were identical to the known sequence in the gene library, and no mutation was observed. Thus, the prokaryotic expression vector of human anti-TLR4 Fab fragment (pDuet-TLR4-Fab01) has been constructed.

### Expression and purification of hTLR4-Fab in *E*. *coli* BL21

**Fig 2:**
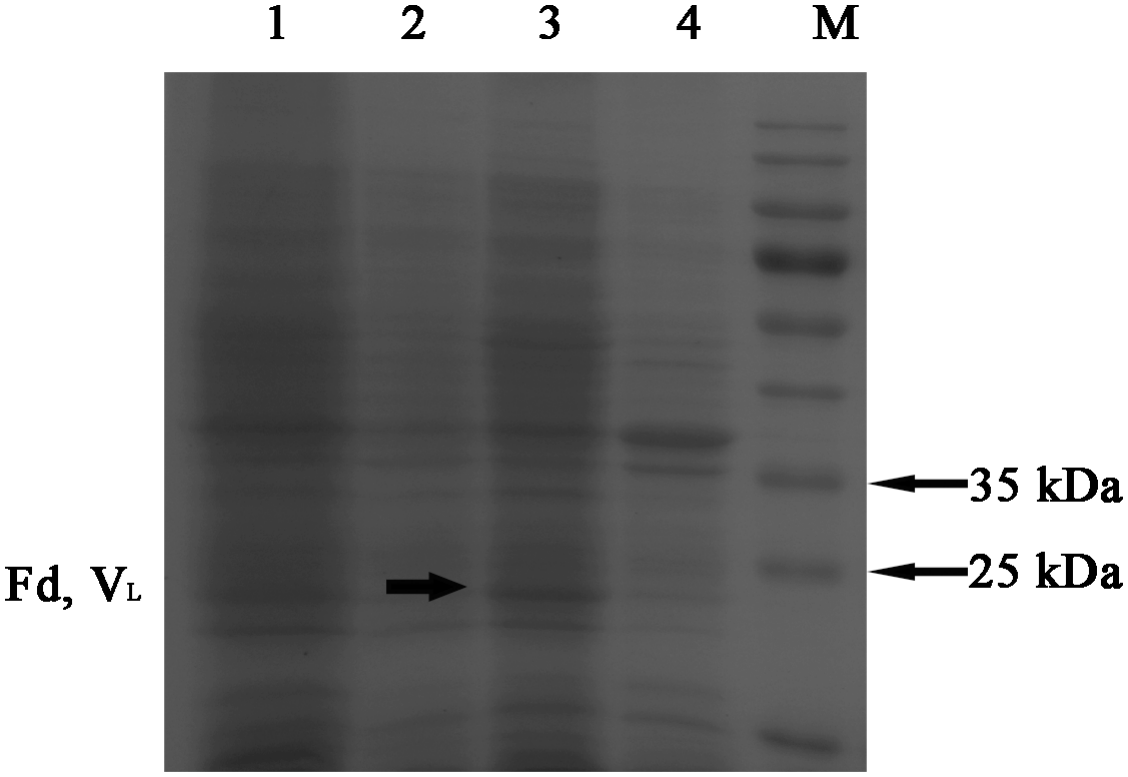
Expression of the recombinant vector pDuet-hTLR4-Fab01. Coomassie blue staining showed that the recombinant vector was expressed during the induction period. lane 1, whole lysate of pDuet-hTLR4-Fab01 transfected *E*. *coli*; Lane 2, lysate of untransfected *E*. *coli* BL21, as a negative control; lane 3, supernatant of sonicated lysate of pDuet-hTLR4-Fab01 transfected *E*. *coli*; lane 4, sediment of sonicated lysate of pDuet-hTLR4-Fab01 transfected *E*. *coli*; lane M, protein marker. All strains were induced by IPTG overnight.

The pDuet-TLR4-Fab01 plasmid was transfected into *E*. *coli* BL21, and induced at 37°C with 1 mmol/L of IPTG overnight. The recombinant bacteria expressed a majority of proteins at approximately 27 kDa, which were detected by SDS-PAGE with Coomassie brilliant blue staining. Due to the weight of the heavy chain Fd was similar to that of the light chain L, the two bands overlapped on the SDS-PAGE gel. While the band of the ultrasonic supernatant was brighter than that of the ultrasonic sediment (Fig 2).

**Fig 3:**
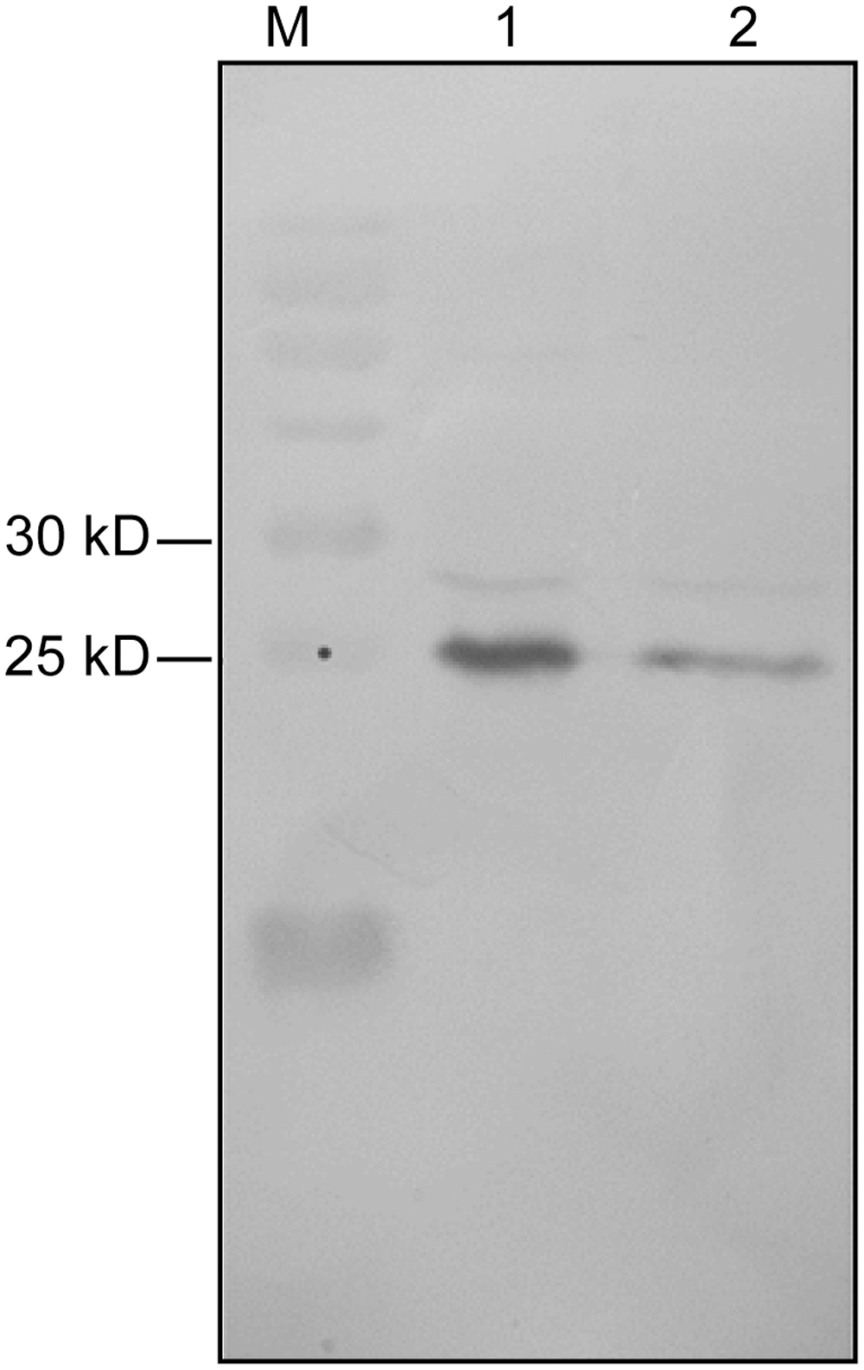
Expression and purification of hTLR4-Fab01 in *E*. *coli* BL21. HRP-conjugated goat anti-human Fab specific antibody was used to detect Fab expression in Western blotting. Lane 1, supernatant of sonicated lysate of pDuet-hTLR4-Fab01 transfected *E*. *coli* induced by IPTG overnight; lane 2, sediment of sonicated lysate of pDuet-hTLR4-Fab01 transfected *E*. *coli* induced by IPTG overnight; lane M, protein marker.

Considering to the presence of recombinant protein, the lysate was further analyzed by western blotting with HRP-conjugated goat anti-human Fab specific antibody. Western blot results showed an obvious band at approximately 27 kDa, which was not found in the sample without IPTG induction, and the band of the ultrasonic supernatant was brighter than that of the ultrasonic sediment (Fig 3).

**Fig 4:**
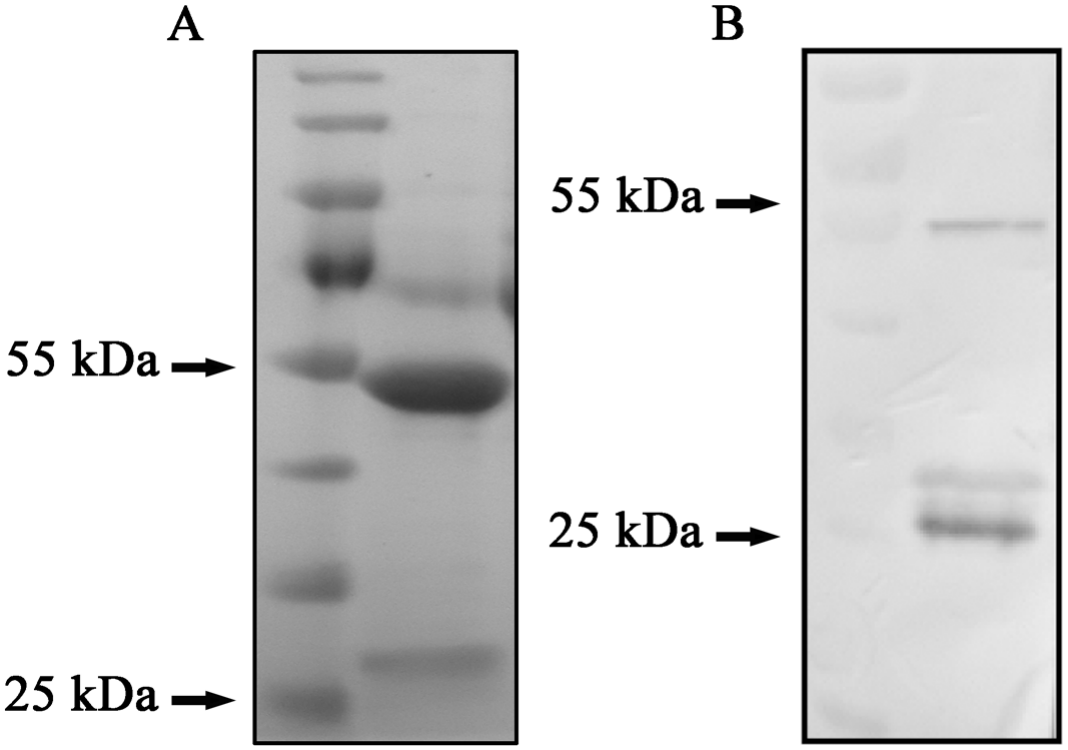
Detection of the hTLR4-Fab01 by native-PAGE and Western blotting. (A) Coomassie blue staining showed that the hTLR4-Fab01 was expressed, the heavy chain Fd and light chain L were linked together. (B) HRP-conjugated goat ant-human Fab specific antibody was used to detect the heavy chain Fd and light chain L of the hTLR4-Fab01 was expressed and separated in Western blotting.

Compared with SDS-PAGE electrophoresis, native-PAGE greatly reduces the probability of protein denaturation. Gel electrophoresis produced two bands, the brighter band at 55 kDa and less one at 25 kDa, the western blot analysis exhibited that the protein was specifically bound by HRP-conjugated goat anti-human Fab specific antibody (Fig 4).

**Fig 5:**
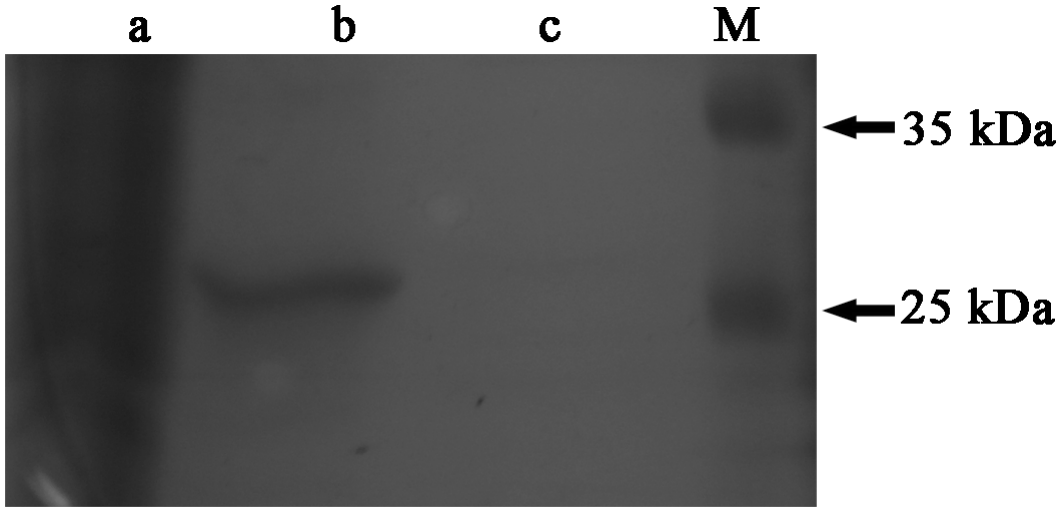
The efficiency of hTLR4-Fab01 purification. Lane 1, whole lysate of pDuet-hTLR4-Fab01 transfected *E*. *coli* induced by IPTG overnight; lane 2, supernatant of sonicated lysate of the pDuet-hTLR4-Fab01 transfected *E*. *coli* was purified by the His-trap Lambda Fab Select column; lane 3, supernatant of sonicated lysate after purification; lane M, protein marker.

Due to the light chain of the recombinant protein is the lambda chain, the His-trap Lambda Fab Select column was chosen to purify the Fab fragment. The purity of the target protein was above 95% after purification, and the concentration of the hTLR4-Fab01 was about 0.7 mg/ml (Fig 5). Taken together, the hTLR4-Fab01 was successfully expression at a high concentration, which was sufficient for the examination of its affinity and bioactivity.

### The hTLR4-Fab01 specifically binds the TLR4 antigen

**Fig 6:**
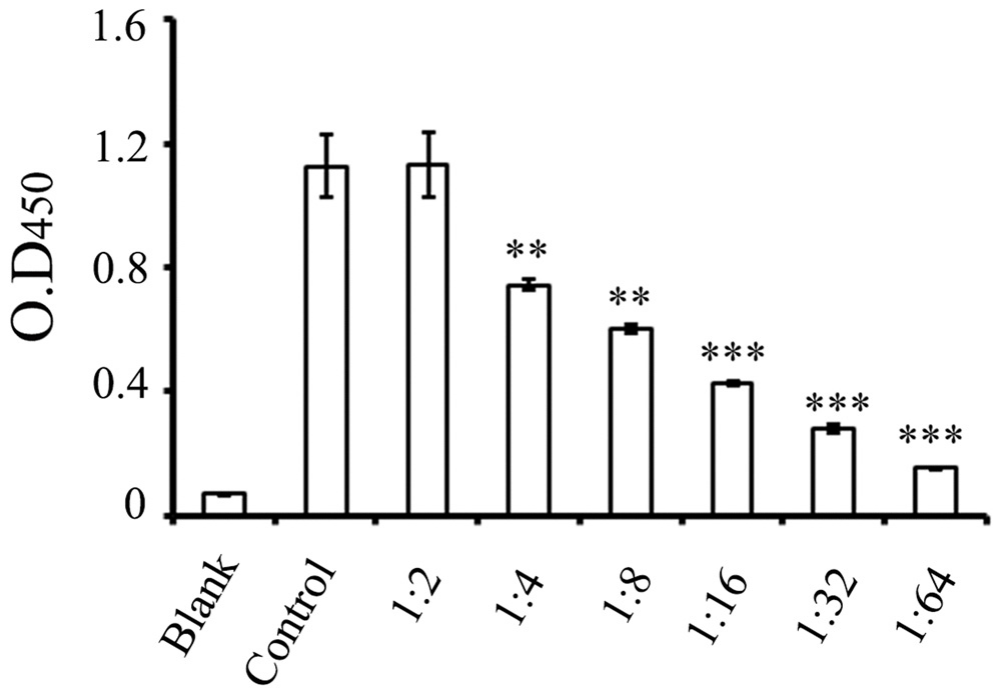
The hTLR4-Fab01 specifically binds the TLR4 antigen. Ninety-six-well plates were pre-coated with recombinant human TLR4, 10 μg/ml. Serial concentrations of the hTLR4-Fab01 were used as the primary antibody. HRP-conjugated anti-human Fab specific antibody was used as the secondary antibody. Commercial TLR4 antibodies were used as positive control. The absorbance was read at 450 nm after color development. Experiments were performed in triplicate. Data are shown as mean ± SD (n = 3, ** p < 0.01, *** p<0.001 compared to positive control)

To test whether the hTLR4-Fab01 could bind to recombinant human TLR4 specifically. The ELISA assay was carried out with different concentration of hTLR4-Fab01. We found that the purified protein could effectively bind to recombinant human TLR4 in a dose-dependent manner. While the control antibody anthrax chimeric Fab with different antigen specificity could not bind to the recombinant human TLR4. Furthermore, the hTLR4-Fab01 concentration was diluted from 0.35 mg/ml to 0.022 mg/ml, the absorbance value at 450 nm decreased from 1.1255 to 0.426 (Fig 6).

**Fig 7:**
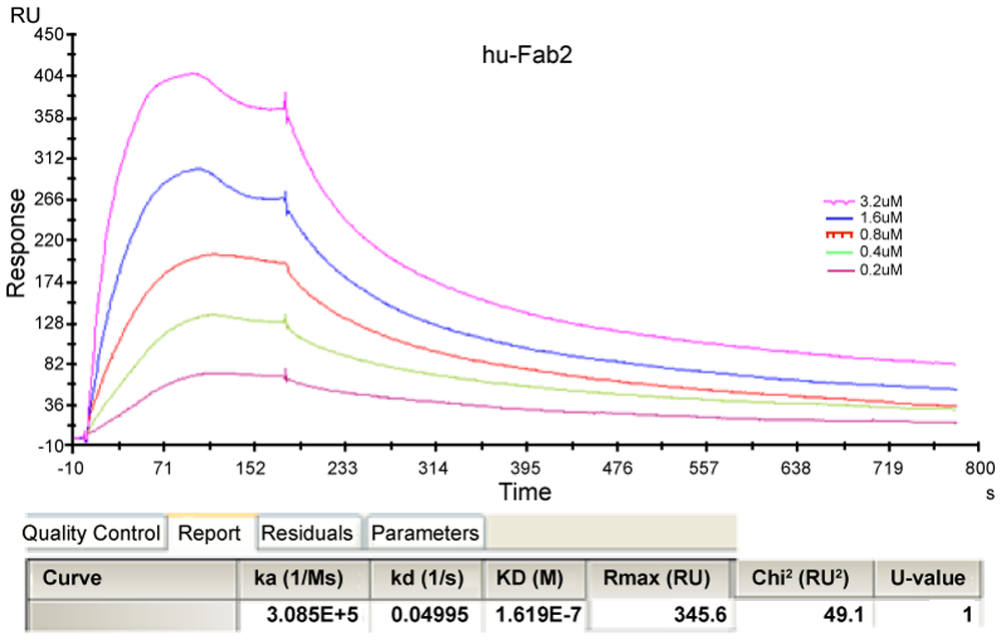
Affinity measured by Biacore X100. The sample was diluted with buffer solution to 30 μg/ml, and then treated with a running buffer containing different concentrations of hTLR4-Fab01. Results were analyzed using the Biacore X100 software.

To further analyze the binding ability of hTLR4-Fab01, the affinity constant was calculated using the following formula: affinity constant (KD) = dissociation constant (Kd) ÷ binding constant (Ka), which reflected the degree of antigen-antibody reaction; the smaller of the value, the stronger of its binding affinity [26]. Results from the Biacore X100 SPR analysis displayed that the affinity of hTLR4-Fab01 was about 1.619× 10^−7^ (Fig 7). Taken together, these results demonstrated that hTLR4-Fab01 could specifically binds to hTLR4.

### The hTLR4-Fab01 bind to the cell-surface TLR4 antigen

**Fig 8:**
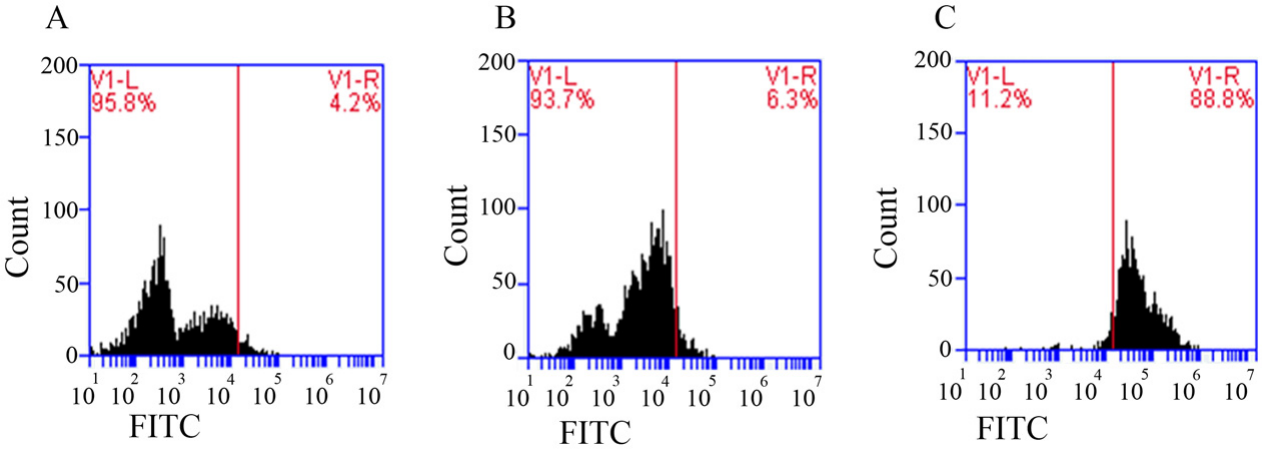
The hTLR4-Fab01 bind to the cell-surface TLR4 antigen. THP-1 cells were treated(C) or untreated (B) with hTLR4-Fab01 at 4°C for 1 h, FITC-conjugated goat anti-human Fab specific antibody was incubated with THP-1 cells for 1 h in the dark at 37°C. (A), control. The cells were determined by flow cytometry.

Further analysis about the binding specificity of hTLR4-Fab01 was performed by Flow cytometry using TLR4-positive THP-1 cells. Flow cytometry analysis showed that the population of hTLR4-Fab01 treated THP-1 cells was separated from unbinding cells by fluorescent intensity, whereas no obvious difference was observed without hTLR4-Fab01 treated cells (Fig 8), suggesting that hTLR4-Fab01 binds to TLR4-positive cells efficiently. These data demonstrate the high affinity and specificity of hTLR4-Fab01 was isolated.

### The hTLR4-Fab01 inhibits LPS-induced proinflammatory cytokines production

**Fig 9:**
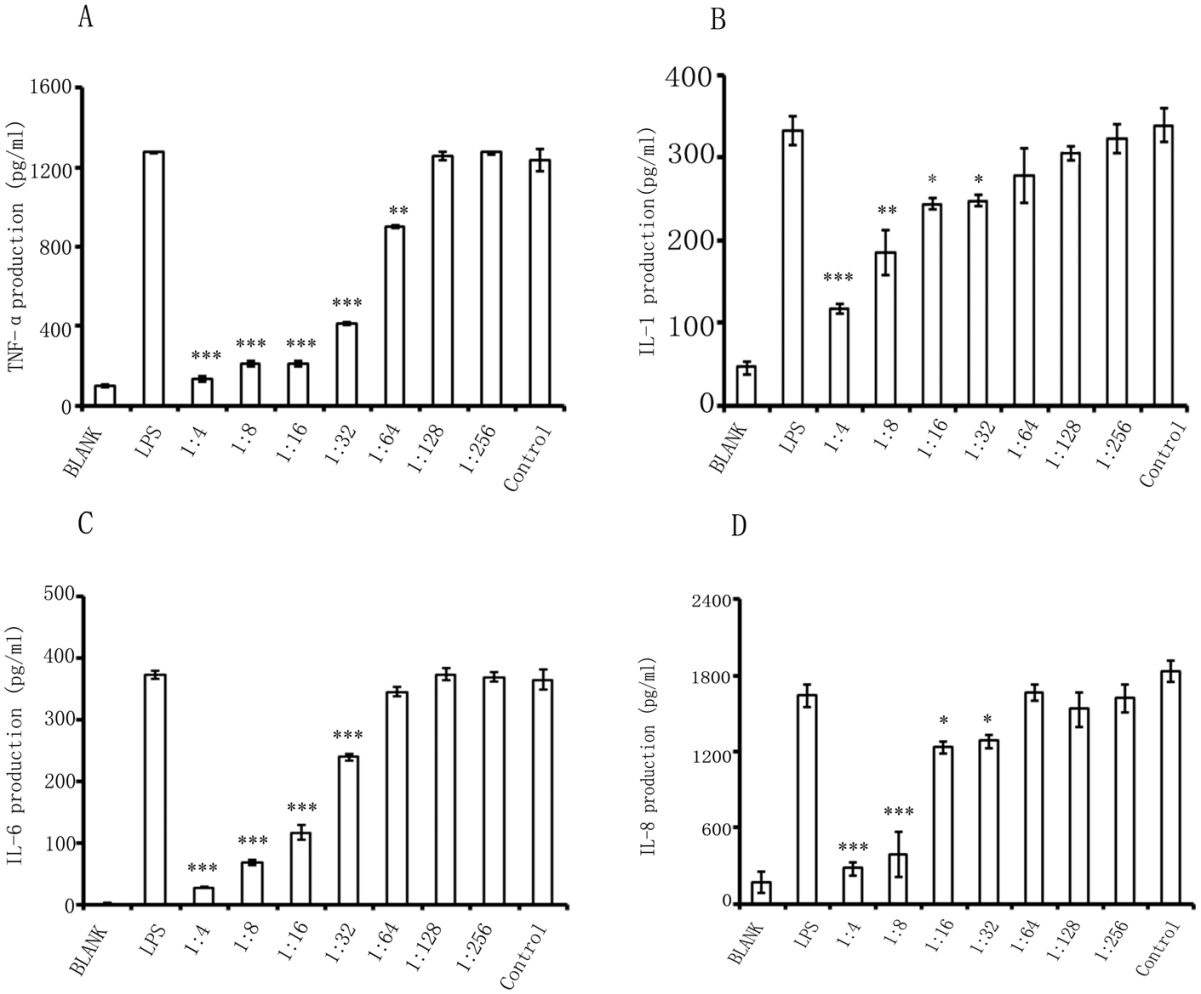
The hTLR4-Fab01 inhibits LPS-induced secretion of pro-inflammatory cytokines in vitro. Human macrophage cells were incubated with different concentrations of human anti-TLR4 antibody Fab for 2 h. Anthrax chimeric Fab antibody was used as the negative control. Cells were stimulated with LPS (100 ng/ml) for 4 h. Secreted TNF-α (A), IL-1β(B), IL-6 (C), and IL-8 (D) were quantified by ELISA. Experiments were performed in triplicate. Data are shown as mean ± SD (n = 3, * p < 0.05, ** p< 0.01, *** p<0.001 compared to LPS group)

We were intended to examine whether specific binding of the TLR4 receptor by the hTLR4-Fab01 could influence LPS-induced proinflammatory cytokines production. The THP-1 transformed macrophages were incubated with the hTLR4-Fab01 then stimulated with LPS, the supernatant was collected to detect the pro-inflammatory cytokines. We found that hTLR4-Fab01 could obviously neutralize TLR4 activation in macrophages, such as secretion of TNF-α, IL-1β, IL-6, and IL-8 was significantly inhibited. To provide further evidence, different concentration of hTLR4-Fab01 were incubated with macrophages. We found that hTLR4-Fab01 could effectively block LPS-induced proinflammatory cytokines production in a dose-dependent manner. (Fig 9). Thus, the results implied that the hTLR4-Fab01 had the fully capacity to neutralize LPS induced TLR4 activation in vitro.

## Discussion

Humanized antibodies are mostly potential for clinical diagnosis and treatment in lots of diseases, which are constructed by different DNA recombination technologies. Meanwhile, the phage display technology possess the advantage of being inexpensive and efficient. We have previously constructed a fully human Fab phage library [19]. Furthermore, smaller antibody fragments (Fabs or scFvs) can be comprised with phage display. Previous results show that these fragments preserve high efficiency in penetrating into the targeted tissue with high concentration and validity [27].

In this study, we exploited the human Fab phage library to construct an active human anti-TLR4 Fab fragment (hTLR4-Fab01) that could recognize recombinant TLR4 protein. The biopanning strategy with the repeated panning with coated recombinant TLR4 protein in 96-well plates ensured the enrichment of specific TLR4 binding phage. After fifth rounds of panning, one of selected 80 candidate phage clones exhibited strong positive signal, and showed the most specifical binding by ELISA. The prokaryotic expression vector pDuet-TLR4-Fab01 was successfully constructed. This fascinating approach ensures that the two different chains are fairly expressed, efficiently dimerized and formation of active hTLR4-Fab01, which guaranteed production of stable antibody fragments [28–29]. Western blotting and SDS-PAGE with Coomassie blue staining confirmed the highly expression of hTLR4-Fab01. Furthermore, the specifical binding of hTLR4-Fab01 to TLR4 on the surface of THP-1 transformed macrophages were confirmed by Flow cytometry. Thus these results demonstrate that the antibody engineering process did not change the specificity of the human anti-TLR4 Fab.

TLRs are fundamental in mediating the pro-inflammatory response, and TLR4 has been recognized as the most critical toll homolog which mainly responds to LPS [30]. The activation of KCs, HSC, and hepatocytes by LPS contributes to hepatic fibrosis [31]. TLR4 signaling is expressed on activated KCs, which primarily conduct the signaling pathways of HSC. Furthermore, TLR4 enhances KCs activation through TNF-α and IL-6 signaling promoting fibrogenesis [12]. Therefore, TLR4 is predicated to be involved in pathogenesis of hepatic fibrosis. In this study, we found that the production of TNF-α, IL-1β, IL-6 and IL-8 were down-regulated in hTLR4-Fab01 treated macrophages compared with control Fab treated group. The down-regulation of these cytokines might be beneficial in preventing KCs activation. To the best of our knowledge, this is the first study to report the anti-inflammatory of hTLR4-Fab01 antibody, which may have a better protective effect against the progress of hepatic fibrosis.

It remains to be seen how the interaction between hTLR4-Fab01 and TLR4 could block the downstream signaling transduction. One possibility is that hTLR4-Fab01 directly binds to the site of LPS binding in TLR4 and simply prevent the LPS recognition. In this case, less LPS is able to access the binding site while in the presence of hTLR4-Fab01, as reflected by lower proinflammatory cytokines production (Fig 9). Additional studies should be performed to clarified the mechanism and investigate its biological effects in vivo.

In conclusion, we present the preparation and characteristics of a human anti-TLR4 Fab fragment (hTLR4-Fab01), which could specifically bind to TLR4 with high affinity, neutralize LPS-induced TLR4 activation and reduce the production of proinflammatory cytokines in macrophages. Finally, our study provides a novel and promising strategy for prevention and treatment of hepatic fibrosis.
